# *Panagrolaimus superbus* tolerates hypoxia within Gallium metal cage: implications for the understanding of the phenomenon of anhydrobiosis

**DOI:** 10.21307/jofnem-2020-046

**Published:** 2020-05-18

**Authors:** Danyel Fernandes Contiliani, Yasmin de Araújo Ribeiro, Vitor Nolasco de Moraes, Tiago Campos Pereira

**Affiliations:** 1 Department of Biology, FFCLRP, University of São Paulo, Ribeirão Preto, Brazil; 2 Graduate Program of Genetics, FMRP, University of São Paulo, Ribeirão Preto, Brazil

**Keywords:** Anhydrobiosis, Hypoxia, Metal, *Panagrolaimus superbus*, Resistance

## Abstract

*Panagrolaimus superbus* nematodes are able to tolerate desiccation by entering into a peculiar state of suspended animation known as anhydrobiosis. When desiccated, anhydrobiotic organisms are also able to tolerate other physical stresses, as high and low levels of temperature and pressure. Here, we decided to investigate the tolerance of desiccated *P. superbus* to an unprecedented double stress – hypoxia within 99.99% Gallium (Ga) metal cage. The authors observed that regardless of the external relative humidity, desiccated *P. superbus* tolerated 7 d confined within the metal cage, displaying no negative effects on its survival and population growth rates over 40 d. The results evidence that anhydrobiosis also renders nematodes tolerant to otherwise lethal concentrations of Ga, in an oxygen-poor environment; thus, expanding its polyextremotolerance profile.

Nematodes have adapted to live in different ecosystems (marine, freshwater, and terrestrial environments) and hosts (as parasites), with a cosmopolitan scattered pattern, inhabiting tropical, temperate and sub-Arctic soils, playing important roles in biogeochemistry (van den Hoogen et al., 2019). Importantly, many of these organisms are plant-parasitic entities that lead to drastic impacts on crops worldwide; thus, resulting in global annual losses around $125 billion (Barker et al., 1994; Crow et al., 2000; Chitwood, 2003). Some examples are the soybean-parasitic nematodes *Heterodera glycines*, the root-knot nematodes *Meloidogyne incognita*, and the potato-parasitic nematodes *Globodera rostochiensis*.

In nature, some organisms exhibit a singular survival strategy to droughts known as anhydrobiosis (life without water) − a very stable state of suspended animation into which some species are able to enter when subjected to desiccation, which induces substantial water loss, resulting in a cellular water content of less than 0.1 g g^−1^ dry mass and metabolism ceases (Tunnacliffe and Lapinski, 2003). This phenomenon is reported in bacteria, fungi, plants, and animals (Tunnacliffe and Lapinski, 2003), including some nematodes species, such as *Aphelencus avenae* (Madin and Crowe, 1975), *Ditylenchus dipsaci* (Perry, 1977), *Panagrolaimus superbus* (Aroian et al., 1993; Shannon et al., 2005), among others.

Notoriously, in the dry state, anhydrobiotic organisms exhibit polyextremotolerance, i.e. tolerance to harsh conditions for life, such as high and low temperatures (from −273°C to +151°C) (Rahm, 1923; Becquerel, 1950); ionizing radiation (Keilin, 1959); high hydrostatic pressures (Seki and Toyoshima, 1998); vacuum (Rebecchi et al., 2009); and hyperacceleration (Souza and Pereira, 2018). Biological activity is fully restored after rehydration. Anhydrobiosis is an important phenomenon in nematodes since it may allow the long-term survival of plant-parasitic and/or of free-living nematodes species during droughts, which may tend to be more intense and frequent in the light of climate change.

An unresolved question is whether, in fact, metabolism is completely interrupted during anhydrobiosis (Wharton, 2015). In order to answer this question, Barrett (1982) fed nematodes with radiolabeled glucose and searched for metabolic products during the period of desiccation. Similarly, Örstan (1998) stored desiccated bdelloid rotifers inside argon gas chambers and reported their survival even after several periods in the absence of oxygen – necessary for metabolism. Lastly, computer-based data revealed that the remaining water contents in desiccated colonies of *Nostoc commune*, bacterial cells, *Artemia* cysts, and plant seeds (i.e. less than 0.1 g g dry mass^−1^) are insufficient to sustain an active metabolism (Clegg, 1978, 1986; Potts, 1994).

Gallium (Ga) is a soft metal categorized as a post-transition metal, sharing some physicochemical characteristics with Aluminium, Indium, and Thalium. In particular, Ga has low melting point (29.76°C) (Jefferson Lab, 2003) and is slowly oxidized (Moskalyk, 2003), when compared to other metals. Previously, we have shown that desiccated *P. superbus* nematodes are tolerant to temperatures up to 50°C (de Souza et al., 2017). This observation raised the possibility of immersing desiccated nematodes within liquid Ga (at 30°C) to assess their tolerance to such abiotic stress.

Therefore, in the present study, our objective was to investigate the tolerance of desiccated (in anhydrobiosis) and hydrated (living) *P. superbus* nematodes to Ga metal.

## Materials and methods

### Nematode maintenance

*Panagrolaimus superbus*, first isolated from Surtsey Island (Iceland) (Sohlenius, 1988), was maintained in the dark, at 20°C, on Nematode Growth Medium (NGM) agar plates and fed with a layer of *Escherichia coli* (OP50 strain).

### Population synchronization

Mixed populations of *P. superbus* (i.e. containing all developmental stages) were subjected to synchronization using the bleaching protocol proposed by Stiernagle (2006). Briefly, these populations were exposed to a bleaching solution (NaOH 1 M and NaClO 40%) for 8 min, disintegrating all the worms, only eggs remained. The reaction was stopped by three consecutive washes with M9 buffer, intercalated with centrifugations at 2,000 × g for 30 sec. All the remaining eggs were deposited on agar NGM plates containing *E. coli* OP50 and maintained in the dark at 20°C. After 36 hr, plates were rinsed with M9 buffer to collect L2 larvae, which were subjected to the desiccation process.

### Desiccation challenge

Nematodes were subjected to desiccation assay according to Shannon et al. (2005). L2 larvae were immobilized on 0.45 μm Supor filter membranes (Sigma Aldrich) by vacuum filtration with a Sartorius funnel (*n* = 200 worms per membrane), which were stored in 1.5 ml open test tubes and then subjected to sealed chambers under the following conditions: pre-conditioning in 98% relative humidity (RH) for 24 hr over a saturated copper sulphate solution and desiccation in 10% RH for 24 hr on dry silica gel.

### Immersion within Gallium

**Figure 1:**
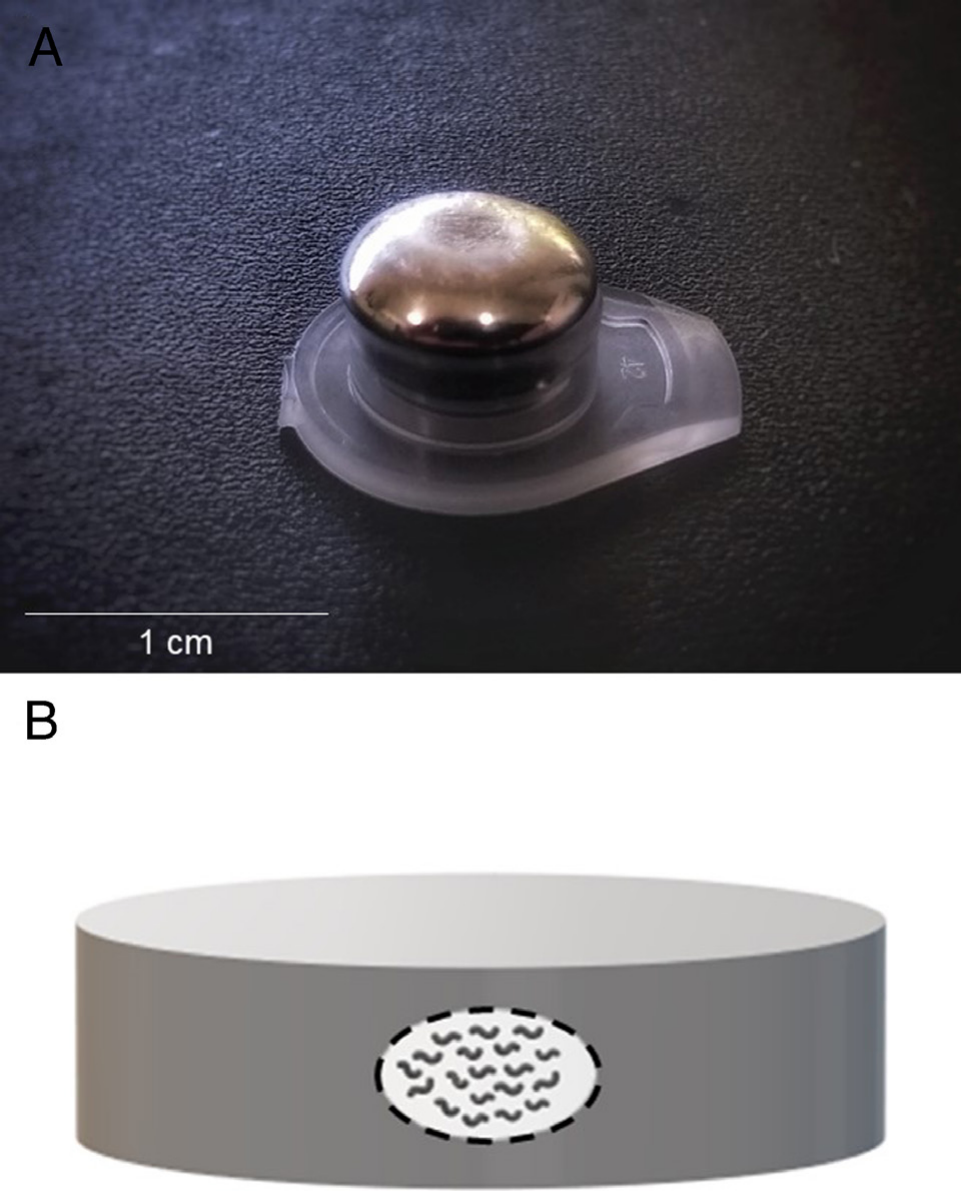
Gallium confinements. A: Gallium metal-containing test tube caps, B: metallic confinement (gray) scheme showing the membranes (white) with nematodes.

Three membranes containing around 600 desiccated L2 larvae were placed in test tube caps containing 100 μl solid Ga 99.99% (Sigma Aldrich). Subsequently, 180 μl liquid Ga 99.99% (Sigma Aldrich), at 50°C, was added to the test tube caps fully covering the membranes (Fig. 1A, B). To check the ability of metal Ga in blocking external moisture, Ga cages containing nematodes were exposed to different levels of relative humidity. Test tube caps were separated into five groups, in the following conditions: negative control 1 (NC1) – membranes with desiccated worms, without Ga, kept at 10% RH; Ga treatment 1 (GT1) – membranes with desiccated worms, covered by Ga and kept at 10% RH; hydrated control (HC1) – membranes with hydrated worms, covered by Ga, kept at 100% RH; negative control (NC2) – membranes with desiccated worms, without Ga, kept at 99% RH; Ga treatment 2 (GT2) – membranes with desiccated worms covered by Ga, kept at 99% RH. All groups were maintained under these same conditions for 7 d, at 20°C in the dark.

### Survival assay

To investigate nematode viability after treatments, we performed worm survival assay using a modified version of Krause et al. (1984) protocol, which was first used for isolated cells but has already been successfully used for nematodes by Evangelista et al. (2017). Briefly, solid Ga blocks (placed within test tube caps) (Fig. 1) were heated at 50°C for 15 min, in order to melt the metal cages. Subsequently, most of the liquid metal was collected from the tube caps by using a micropipette. Finally, we used tweezers to collect the membrane from the residual liquid Ga inside the tube caps. All membranes were deposited in 1.5 ml open test tubes, which remained in 99% RH for 24 hr, for pre-rehydration. Then, one membrane per treatment (*n* = 200) was transferred to other 1.5 ml test tubes containing 1 ml erythrosine B dye (0.4% w/v in M9 buffer). After 1 hr, samples were washed three times with M9 buffer, to remove the dye excess. Totally and partially dyed worms were scored as dead.

### Population growth analysis

Two membranes per treatment (*n* = 400) were submerged in 1 ml M9 buffer and the worms were rehydrated. About 100 worms were placed on new NGM plates with *E. coli* OP50 and maintained at 20°C in the dark. Population growth was measured after 20 d, as well as the survival percentages, and about 100 worms of each treatment were placed on new NGM plates with *E. coli* OP50 for more 20 d (until day 40). Population growth was determined as follows: [(number of worms − output) × (final survival percentage)] divided by [(number of worms − input) × (initial survival percentage)].

### Data analysis

All experiments were performed in biological and technical triplicates with around 100 individuals. Mean values and standard deviations were generated for each experimental group. Shapiro–Wilk’s and Levene’s tests were first performed (*P* > 0.05) and, then, statistical analyses were performed using one-way ANOVA and Tukey’s *post hoc* test. Statistical differences were considered significant at *P* < 0.05.

## Results

### Survival assay

**Figure 2:**
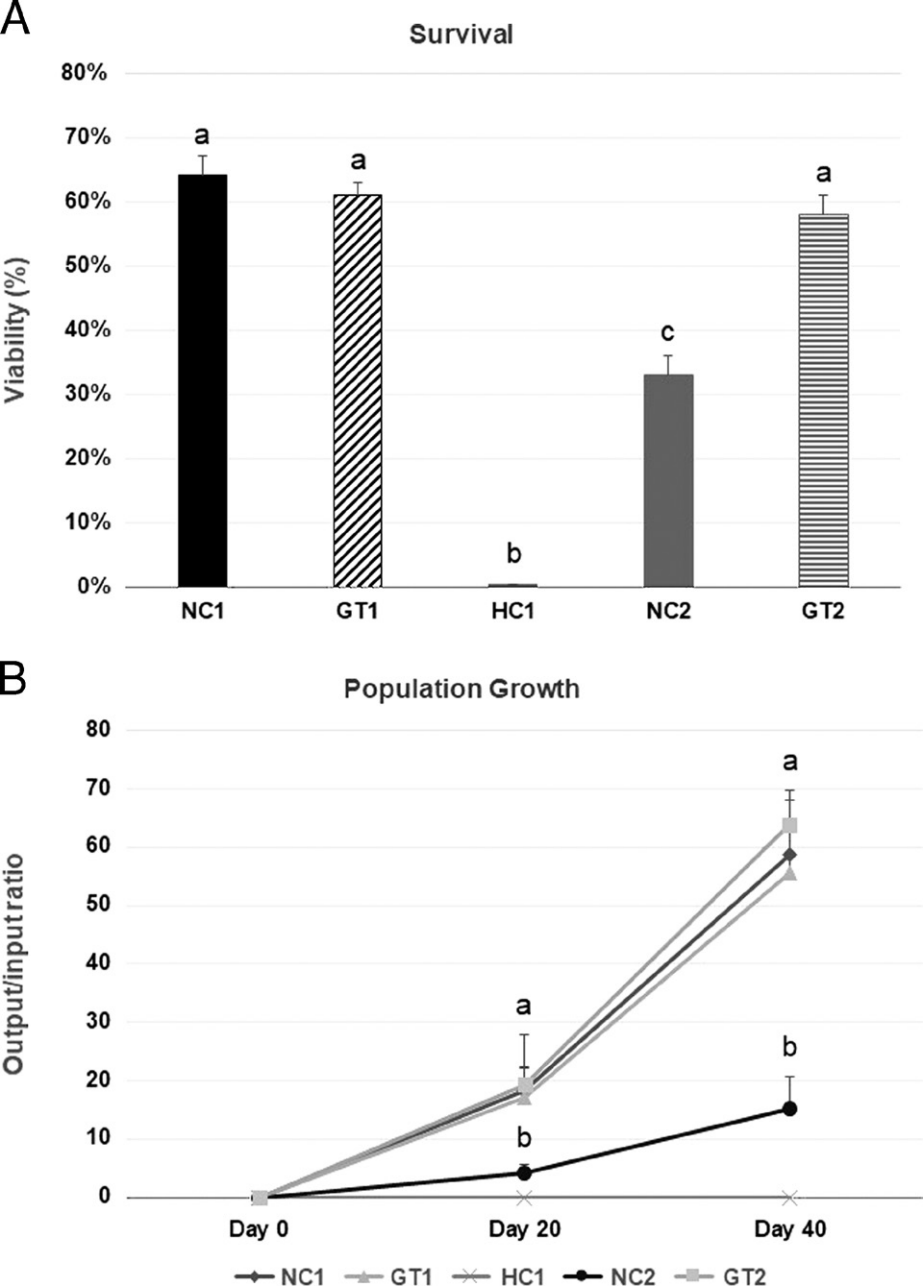
*P. superbus* tests after 7 d of Ga immersion. A: Survival (percentage) of L2 nematode larvae, B: population growth (output/input ratio) over 20 and 40 d after treatments. Legends: negative control 1 (NC1) – membranes with desiccated worms, without Gallium, in 10% RH; Gallium treatment 1 (GT1) – membranes with desiccated worms covered by Gallium in 10% RH; hydrated control (HC1) – membranes with hydrated worms covered by Gallium in 100% RH; negative control (NC2) – membranes with desiccated worms, without Gallium, in 99% RH; Gallium treatment 2 (GT2) – membranes with desiccated worms covered by Gallium in 99% RH. Different letters (a and b) means statistical difference (*P* < 0.05) between treatments.

Desiccated *P. superbus* survival percentages (Fig. 2A) after 7 d fully immersed in Ga confinements did not show statistically significant differences compared to NC1. The experimental group GT2 (66%) displayed differentially (*P* < 0.05) higher survival percentages compared to NC2 group (31%), but statistically equivalent to NC1 and GT1 groups (73 and 72%, respectively). Lastly, HC1 group showed no survival (0%) after immersion in Ga for 7 d.

### Population dynamics

Likewise, NC1, GT1, and GT2 population growth values (Fig. 2B) did not significantly (*P*  > 0.05) differ within 40 d (58.8, 55.6, and 63.9-fold at day 40, respectively). Over 40 d, NC2 group showed statistically significant lower population growth (15.3-fold at day 40) compared to all experimental groups. No population growth was observed in group HC1.

## Discussion

Under hydrated settings (i.e. group HC1), we show that the *P. superbus* is unable to survive a seven-day confinement in Ga-rich due to metal toxicity, hypoxia, and/or food shortage, resulting in natural death by starvation. Conversely, desiccated worms that were kept in exposure to external moisture in the absence of Ga (i.e. group NC2) gradually rehydrated, and thus were challenged by food shortages.

On the other hand, the NC1 group remained desiccated in a Ga-free dry condition (10% RH) and kept in suspended animation ensuring a high survival rate, which is expected since *Panagrolaimus* sp. are able to remain viable even after 8 yr in desiccation (Aroian et al., 1993). We also showed that the desiccated GT1 group inside Ga, which was also kept in a dry condition (10% RH), displayed high tolerance to metallic stress due to anhydrobiosis (equivalent survival percentages to its control group, NC1). Lastly, since the desiccated GT2 group inside Ga also had a high survival percentage even at 99% RH, we showed that Ga cages are able to isolate nematodes from external moisture; otherwise, nematodes would be rehydrated over time and, consequently, would starve to death.

Therefore, our results show for the first time, the high tolerance of desiccated *P. superbus* nematodes after full immersion in 99.99% Ga metal cage, with no negative effects on its survival and population growth. It suggests the operation of intrinsic strategies of anhydrobiotic repair machinery against a hypoxic/anoxic and metal-rich condition, such as hypoxia inducible factors (Sorathia et al., 2019) and, possibly, metalloproteins (Vijver et al., 2004; Li et al., 2016). Importantly, the cuticle also probably plays an important role in the protection against the metal.

Since Ga-based semiconductors production has attracted attention of the microelectronics industry (Flora, 2000), an increase in Ga concentration in soil has been evidenced (Angelone and Bini, 1992; Kabata-Pendias and Pendias, 1984) and possibly correlated with its increasing global consumption (Jaskula, 2018), which may result in toxic levels of Ga in terrestrial and aquatic environments, threatening the local biodiversity. Therefore, we should encourage studies related to Ga toxicity and biological tolerance to heavy metals.

Although Ga toxicity has not been widely explored, its acute and chronic lethal concentrations (LC_50_) have already been investigated in a few prokaryotic and eukaryotic organisms (Bireg et al., 1980; Lin and Hwang, 1998; Onikura et al., 2005; García-Contreras et al., 2014). Here, we show that more than a half of the desiccated *P. superbus* population tolerated an exposure to 5.9 × 10^6^ mg/L of liquid Ga, a concentration which is 6 × 10^4^ times higher than the 96 hr-LC_50_ revealed for the fish *Cyprinus carpio* (Betoulle et al., 2002), for comparison.

Heavy metal tolerance can be observed in different nematodes species. Metals may stimulate sensorial apparatus (e.g. metal-ion receptors) (Sambongi et al., 1999) which, subsequently, trigger physiological responses (e.g. pharyngeal pumping ceasing) (Jones and Candido, 1999). In addition, as reported here, this tolerance can be strongly enhanced by anhydrobiosis. More importantly, some plant-parasitic nematodes, such as *D. dipsaci* (Perry, 1977), also exhibit anhydrobiosis as a survival strategy, which could guarantee similar tolerance mechanisms and render them even more difficult to control for agronomic purposes. Finally, the increased tolerance of desiccated anhydrobiotic nematodes to Ga might result in changes in their abundance in metal polluted areas.

Anhydrobiosis is a singular biological state promoted by desiccation, which renders some organisms tolerant to diverse physicochemical stresses. This phenomenon is especially important in the current scenario of a global warming threat, when droughts tend to occur more frequently. Therefore, understanding the process of anhydrobiosis in nematodes is crucial since several species play important roles in soil and aquatic substrate chemistry, and others impact economically important crops.
